# Dynamical Mechanisms of Interictal Resting-State Functional Connectivity in Epilepsy

**DOI:** 10.1523/JNEUROSCI.0905-19.2020

**Published:** 2020-07-15

**Authors:** Julie Courtiol, Maxime Guye, Fabrice Bartolomei, Spase Petkoski, Viktor K. Jirsa

**Affiliations:** ^1^Aix-Marseille Univ, Inserm, INS, Institut de Neurosciences des Systèmes, 13005 Marseille, France; ^2^Aix-Marseille Univ, CNRS, Centre de Résonance Magnétique et Biologique et Médicale (CRMBM), 13005 Marseille, France; ^3^Assistance Publique-Hôpitaux de Marseille, Hôpital de La Timone, CEMEREM, Pôle d'Imagerie Médicale, CHU, 13005 Marseille, France; ^4^Assistance Publique-Hôpitaux de Marseille, Hôpital de La Timone, Service de Neurophysiologie Clinique, CHU, 13005 Marseille, France

**Keywords:** brain dynamics, brain network model, epilepsy, fMRI, functional connectivity, resting state

## Abstract

Drug-resistant focal epilepsy is a large-scale brain networks disorder characterized by altered spatiotemporal patterns of functional connectivity (FC), even during interictal resting state (RS). Although RS-FC-based metrics can detect these changes, results from RS functional magnetic resonance imaging (RS-fMRI) studies are unclear and difficult to interpret, and the underlying dynamical mechanisms are still largely unknown. To better capture the RS dynamics, we phenomenologically extended the neural mass model of partial seizures, the Epileptor, by including two neuron subpopulations of epileptogenic and nonepileptogenic type, making it capable of producing physiological oscillations in addition to the epileptiform activity. Using the neuroinformatics platform The Virtual Brain, we reconstructed 14 epileptic and 5 healthy human (of either sex) brain network models (BNMs), based on individual anatomical connectivity and clinically defined epileptogenic heatmaps. Through systematic parameter exploration and fitting to neuroimaging data, we demonstrated that epileptic brains during interictal RS are associated with lower global excitability induced by a shift in the working point of the model, indicating that epileptic brains operate closer to a stable equilibrium point than healthy brains. Moreover, we showed that functional networks are unaffected by interictal spikes, corroborating previous experimental findings; additionally, we observed higher excitability in epileptogenic regions, in agreement with the data. We shed light on new dynamical mechanisms responsible for altered RS-FC in epilepsy, involving the following two key factors: (1) a shift of excitability of the whole brain leading to increased stability; and (2) a locally increased excitability in the epileptogenic regions supporting the mixture of hyperconnectivity and hypoconnectivity in these areas.

**SIGNIFICANCE STATEMENT** Advances in functional neuroimaging provide compelling evidence for epilepsy-related brain network alterations, even during the interictal resting state (RS). However, the dynamical mechanisms underlying these changes are still elusive. To identify local and network processes behind the RS-functional connectivity (FC) spatiotemporal patterns, we systematically manipulated the local excitability and the global coupling in the virtual human epileptic patient brain network models (BNMs), complemented by the analysis of the impact of interictal spikes and fitting to the neuroimaging data. Our results suggest that a global shift of the dynamic working point of the brain model, coupled with locally hyperexcitable node dynamics of the epileptogenic networks, provides a mechanistic explanation of the epileptic processes during the interictal RS period. These, in turn, are associated with the changes in FC.

## Introduction

Drug-resistant focal epilepsy is a large-scale brain networks disorder (Bartolomei et al., 2005, 2008, 2017). These networks are typically studied using resting state (RS)-functional connectivity (FC)—the statistical interdependencies in the spontaneous activity between brain regions (Biswal et al., 1995)—which can be performed during interictal periods (Guye et al., 2008; Tracy and Doucet, 2015). The majority of RS-FC studies use fMRI without a precise hypothesis of the organization of the epileptogenic network (Waites et al., 2006; Voets et al., 2014). Various contradictory findings were reported, such as globally increased/decreased connectivity (Pereira et al., 2010; Pittau et al., 2012; Wirsich et al., 2016), or concomitantly increased and decreased connectivity in neighboring networks (Bettus et al., 2009, 2010; Liao et al., 2010; Su et al., 2015). In contrast, most RS-FC studies using magnetoencephalographic (MEG) and electroencephalographic (EEG) recordings indicate predominantly increased connectivity between the regions found to be less connected in fMRI (Bettus et al., 2008; Bartolomei et al., 2013a,b; Schevon et al., 2007; Lagarde et al., 2018; Li Hegner et al., 2018). These discrepancies could be attributed to the different functional aspects captured by each modality or the different RS-FC estimates applied (Ridley et al., 2017). Vigilance fluctuations during the RS-fMRI scans could also be associated with (dynamic) changes in connectivity (Haimovici et al., 2017; Laumann et al., 2017). The effect of interictal epileptiform discharges (IEDs) on the abnormalities of brain networks, such as interictal spikes, is also largely neglected in these studies and might constitute another confounding factor (Bettus et al., 2008; Coito et al., 2016).

In contrast to conventional RS analyses, studies based on brain network models (BNMs) have provided important insights into the mechanisms underlying RS dynamics in health (Ghosh et al., 2008; Deco and Corbetta, 2011) and disease (Cabral et al., 2013; Demirtaş et al., 2017). Spontaneous activity is shaped by the structural connectivity (SC) and the dynamical working point, which refers to the parameters of the model maximally fitting the empirical data. An important common feature of healthy BNMs is the emergence of structured RS fluctuations when the system operates near criticality, namely at the border of a qualitative change in behavior (Deco and Jirsa, 2012; Deco et al., 2013), and shows maximal metastability (i.e., variability of synchronization; Deco et al., 2017a). In epilepsy, the presence of abnormalities even outside the seizure, suggests an alteration of this feature. Hence, we hypothesized that the resting epileptic brain deviates from the healthy critical state. In particular, epileptogenic areas tend to exhibit higher excitability compared with “healthy” regions (Beggs and Plenz, 2003; Monto et al., 2007; Arviv et al., 2016).

Here, we reconstructed 14 epileptic and 5 healthy human BNMs with individual SC and spatial epileptogenicity using The Virtual Brain (TVB; Sanz-Leon et al., 2013, 2015), a neuroinformatics platform that enables biologically realistic modeling and simulation of brain network dynamics using connectome-based approaches and directly linking them to various brain imaging modalities. We developed a population model capable of producing physiological oscillations beyond the epileptiform activity by linearly weighting healthy and epileptiform contributions as ratios of epileptogenicity using the neural mass model Epileptor, which was originally designed to reproduce seizure dynamics (Jirsa et al., 2014). Our proposed approach results from the presumed existence of different zones with varying pathophysiological activity (Lüders et al., 2006), reflecting a gradual change of epileptogenicity. In dynamical systems, discrete onsets of novel behavior are described by bifurcations, which are parametrized by continuous control parameters. The concept of a continuous degree of epileptogenicity has been previously introduced in Jirsa et al. (2014) and applied for the characterization of epileptogenic zones in Proix et al. (2017). Here, we formalized this notion in a region-specific model framework through the extended Epileptor. We then investigated the optimal working point in epileptic BNMs against health through parameter exploration, and tested the effect of *in silico* IEDs and the local shift in the dynamics of epileptogenic regions. This approach allowed us to identify dynamical mechanisms that can explain the empirically observed RS-FC alterations.

## Materials and Methods

### 

#### Patient selection and data acquisition

We selected 14 patients in whom drug-resistant epilepsy had been diagnosed (mean ± SD age, 31.6 ± 11.3 years; 7 females), previously analyzed in Proix et al. (2017). Patients have different types of partial epilepsy accounting for different epileptogenic zone localization and underwent a standard comprehensive presurgical evaluation including structural, functional, and diffusion tensor scans. The findings from this evaluation and details of the patients are summarized in Table 1

**Table 1.** Clinical characteristics of patients with focal epilepsy

Patient	Sex	Duration (years)	Age at onset (years)	Side	Epilepsy type	Surgery outcome (Engel class)	MRI
P1	F	14	8	R	Temporofrontal	III	Anterior TN
P2	F	14	9	L	Occipital	III	N
P3	M	35	7	L	Insular	I	N
P4	F	18	5	L	SMA	I	N
P5	F	16	7	R	Premotor	II	N
P6	M	45	11	R	Temporofrontal	I	FCD Fr
P7	M	5	28	R	Temporal	III	Temporopolar hypersignal
P8	F	18	20	R	Occipital	NO	N
P9	M	11	18	R	Frontal	I	FN (post-traumatic lesion)
P10	F	10	17	R	Temporal	II	HS
P11	M	15	14	R	Temporal	NO	N
P12	M	29	7	R	Temporal	I	Cavernoma
P13	M	28	35	L	Temporal	III	N
P14	F	24	4	R	Occipital	NO	PVH

M, male; F, female; L, left; R, right; SMA, supplementary motor area; NO, not operated; N, normal; FCD, focal cortical dysplasia; Fr, frontal; TN, temporal necrosis; FN, frontal necrosis; HS, hippocampal sclerosis; PVH, periventricular nodular heterotopia.

MRI scans were performed on a Magnetom Verio 3T MR-scanner (Siemens) at the Center for Magnetic Resonance in Biology and Medicine (CRMBM, Marseille, France). T1-weighted anatomical images were acquired with a 3D-MPRAGE sequence (repetition time = 1900 ms; echo time = 2.19 ms; inversion time = 900 ms; voxel size = 1.0 × 1.0 × 1.0 mm^3^), the diffusion MRI images used a diffusion tensor imaging (DTI)-MR sequence (repetition time = 10 700 ms; echo time = 95 ms; angular gradient set of 64 directions; b-weighting of 1000 s/mm^2^; 60 contiguous slices; voxel size = 2.0 × 2.0 × 2.0 mm^3^) and the 20 min resting-state functional MRI images recorded by a BOLD-sensitized EPI T2*-weighted sequence (350 volumes; repetition time = 3600 ms; echo time = 27 ms; voxel size = 2.0 × 2.0 × 2.5 mm; 50 slices; flip angle = 90°). During the resting-state protocol, the patients were asked to stay awake and keep their eyes closed.

Additionally, five healthy control subjects, with no history of neurologic or psychiatric disease who had undergone the same MRI protocol, were also selected to evaluate the dynamical characteristics of subject-specific RS-FC compared with patients with epilepsy and were used in a personalized BNM. All participants signed an informed consent form according to the rules of the local ethics committee (Comité de Protection des Personnes Marseille 2).

#### Data preprocessing

##### Anatomical MRI data preprocessing and structural reconstruction.

The reconstruction of the subjects' individual brain network topography and connection topology within the 3D physical space was performed using an in-house pipeline for automatic processing of multimodal neuroimaging data based on publicly available neuroimaging tools and customized for TVB (https://github.com/the-virtual-brain/tvb-recon). The current version of the pipeline used in this study evolved from a version described previously in Proix et al. (2016).

In short, the pipeline proceeds as follows for each subject: First, the brain anatomy was reconstructed from the T1-weighted images using the *recon-all* command from the FreeSurfer package (version 6.0.0; Fischl, 2012). The T1-weighted images and the generated parcellation volume were then aligned with the diffusion-weighted images (DWIs) using the linear registration tool *flirt* from FSL package (FMRIB Software Library, version 6.0; Jenkinson et al., 2012). The correlation ratio cost function was used for the alignment with 12 degrees of freedom. The tractography was performed using the tools from the MRtrix package (version 0.3.15; Tournier et al., 2012). The fiber orientation distributions were estimated from the DWI using spherical deconvolution (Tournier et al., 2007) by the *dwi2fod* tool with the response function estimated by the *dwi2response* tool using Tournier's algorithm (Tournier et al., 2013). Afterward, 15 million tracts were generated using the *tckgen* tool by a probabilistic tractography algorithm, iFOD2, based on a second-order integration over fiber orientation distribution (Tournier et al., 2010) with the streamlines seeded randomly within the brain volume. Finally, the SC weights matrix *C_ij_* was generated by the *tck2connectome* tool by counting the number of tracts connecting the regions in the Desikan–Killiany parcellation generated by FreeSurfer including 70 cortical regions and 14 subcortical regions (Desikan et al., 2006). No threshold was used to prune weaker edges; however, the BNM is sensitive to connection strength, thereby effectively discarding the effect of smaller weights. All processed data were formatted to facilitate import into TVB, and each *C_ij_* was normalized to unity as $C_{ij}=C_{ij}/\text{max}(C_{ij}(:))$. Note that as the patients have small lesions (in terms of volume), the preprocessing of their MRI scan does not necessitate filling the missing portions of the brain at the lesion site by the brain tissue of the nonlesioned hemisphere homologous (see Falcon et al., 2015 and Aerts et al., 2020, for a detailed description of such procedure).

##### Functional MRI data preprocessing.

RS-fMRI data preprocessing was performed with the FSL *feat* (FMRI Expert Analysis Tool, version 5.0.9; Woolrich et al., 2001) toolbox with standard parameters and not discarding any independent component analysis (ICA) components. The preprocessing steps included the following: discarding the first six images of each scanning run to allow the MRI signal to reach steady state, high-pass temporal filtering to remove slow time drifts (100 s high-pass filter), motion and slice timing correction, spatial smoothing with Gaussian kernel (FWHM = 5 mm), brain extraction, and 12 linear registration to the MNI space.

Then, functional data were registered to the subject's T1-weighted images and parcellated according to FreeSurfer segmentation. By inverting the mapping rule found by registration, anatomical segmentations were mapped to the functional space and average blood-oxygen-level dependent (BOLD) signal time series for each region were generated by computing the spatial mean over all voxel time series of each region. Finally, to limit the effects of physiological noise, the overall time series were temporally low-passed filtered, removing frequencies >0.1 Hz (Cordes et al., 2001).

Note that global regression was not performed as it shifts the distribution of the correlation values of the RS-FC and, in particular, allows the introduction of spurious negative correlations and the underestimation of the true-positive ones (Fox et al., 2009; Murphy et al., 2009). In addition, no additional physiological noise removal was applied, although it is a standard preprocessing procedure when analyzing FC in experimental RS-fMRI studies (Bettus et al., 2009, 2010; Pereira et al., 2010). However, there is also a large number of computational works where this is not the case, even from the same laboratory groups (Schirner et al., 2015, 2018; Deco et al., 2017a; Demirtaş et al., 2017). Having the focus on the novel features of the model and how it can be applied to data fitting, we are less interested in specific activation patterns that could indeed be affected by different acquisition and processing steps, but are more interested in capturing the systematic differences appearing between groups, making the issue of physiological noise of less relevance. Nevertheless, we investigated the effect of head motion and the presence of significant group differences in framewise displacement (FD) for both translational and rotational parameters as movement metric (Power et al., 2012). For the rotational parameters, degrees of arc were converted to millimeters by calculating displacement on the surface of a sphere with a radius of 50 mm. Although epileptic patients tend to move more (mean FD = 0.3605 mm) than healthy control subjects (mean FD = 0.1823 mm) during fMRI acquisition, as observed in previous studies (Lemieux et al., 2007), the difference in mean FD is not significant (two-tailed Wilcoxon rank-sum test: *p* = 0.15; Ridley et al., 2015). This indicates that residual motion-related signals did not make a qualitative change in the forthcoming group results.

##### Individual brain model of resting-state in epilepsy using TVB.

To obtain the BOLD signal during rest, we first virtualized 14 patients with epilepsy and 5 healthy control subjects using The Virtual Brain (version 1.5.6; http://www.thevirtualbrain.org) and simulated the spontaneous neural activity of the brain. TVB is a free open-source neuroinformatics tool designed to aid in the exploration of network mechanisms of brain function and associated pathologies. TVB provides the possibility to feed computational neuronal network models with information about structural and functional imaging data including population [(stereotactic, S)EEG/MEG] activity, spatially highly resolved whole-brain metabolic/vascular signals (fMRI) and global measures of neuronal connections (DTI), for intact as well as pathologically altered connectivity. TVB is model agnostic and offers a wide range of neural population models to be used as network nodes. Manipulations of network parameters within The Virtual Brain allow researchers and clinicians to test the effects of experimental paradigms, interventions (e.g., stimulation and surgery), and therapeutic strategies (e.g., pharmaceutical interventions targeting local areas). The computational environment enables the user to visualize the simulated data in 2D and 3D and to perform data analyses in the same way as commonly performed with empirical data.

Individual virtual human brains were built based on the mutual interactions, in other words, the linear summation as specified in the current subsection, of the local brain region dynamics coupled through the underlying empirical anatomical SC matrix *C_ij_* of each subject obtained from the anatomical and diffusion MRI scans. The interareal connections were weighted by the strength specified in the SC matrix and by a common scaling factor that multiplies all interareal connection strengths. To explore interictal RS network dynamics, we developed a network node model capable of expressing both regionally specific physiological and epileptiform activity. A parameter *p* may scale the relative contribution of each type of activity to the overall node dynamics, as follows:

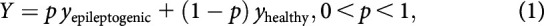
 where *y*_epileptogenic_ and *y*_healthy_ are the respective epileptic and healthy neurons of the population activity *Y*.

**Figure 1.**
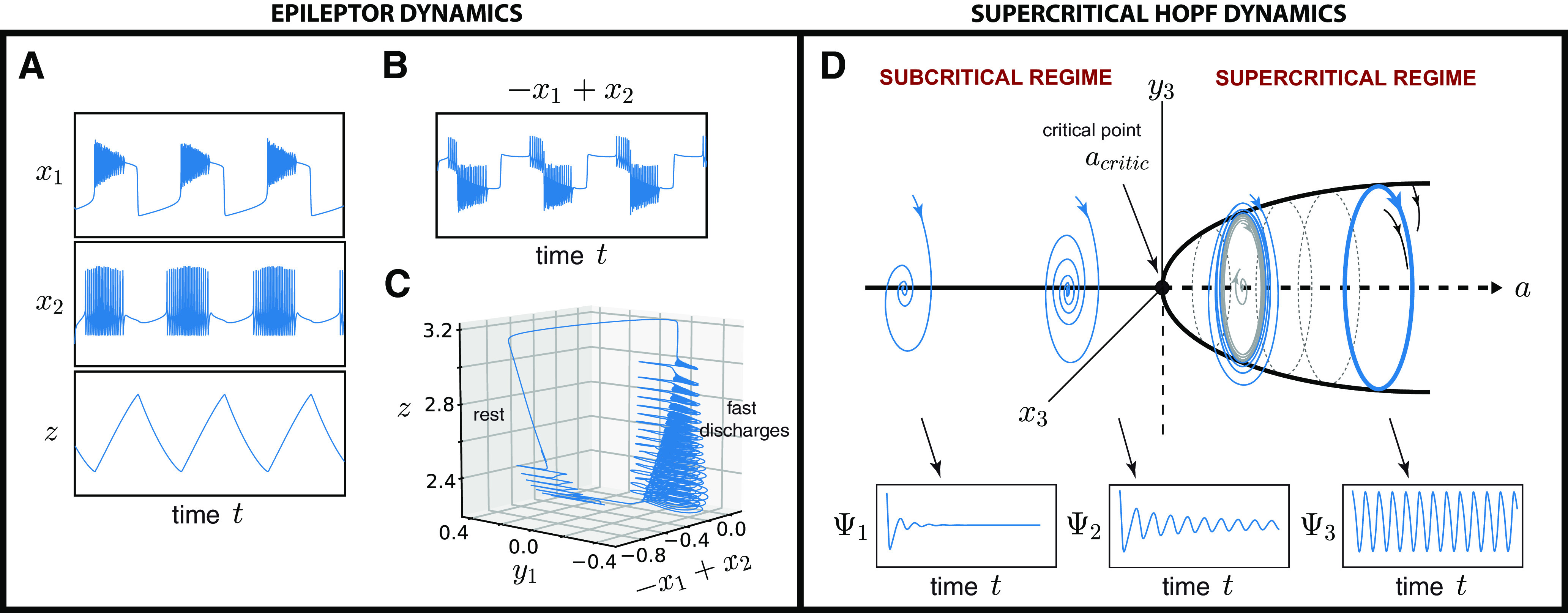
Dynamics of the original Epileptor and supercritical Hopf oscillator model. ***A***, ***B***, Time series of the first *x*_1_ (top), second *x*_2_ (middle), and *z* (bottom) state variables (***A***) as well as the original Epileptor model (***B***; expressed in terms of $-x_{1}+x_{2}$) are plotted. ***C***, The seizure trajectory is approximated in a 3D physical space defined by the state variables (*y*_1_ and$-x_{1}+x_{2}$) and by the slow permittivity variable *z*. ***D***, Depending on the local bifurcation parameter *a*, each region has a supercritical Hopf bifurcation at *a* = *a*_critic_ such that, for $a<a_{\text{critic}}$ the region is in a stable fixed point (subcritical regime) and the system corresponds to a damped oscillatory state, whereas for $a>a_{\text{critic}}$ the region enters in a stable limit cycle and the system switches to an oscillatory state (supercritical regime). The closer a node operates to the critical point, the larger and longer lasting is the oscillation (compared ψ_1_ and ψ_2_). When the critical point is reached, the node intrinsically performs a rhythm of constant magnitude (see ψ_3_).

Previously, regional epileptiform dynamics have been described by the Epileptor model (Jirsa et al., 2014), which was initially designed to realistically reproduce the temporal dynamics of epileptic seizures and also include a slow coupling variable that is responsible for the switching between ictal and interictal states (Fig. 1*B*,*C*). Naively, the novel neural population model could be interpreted as separated into epileptic and healthy neurons, which is possible, but certainly an oversimplification. The gradual parametrization of epileptogenicity is principally a functional differentiation, which may find various mechanistic realizations resulting in the same pathophysiology. The initial Epileptor model comprises three different time scales interacting together and accounting for various electrographic patterns. The fastest and intermediate time scales are two coupled oscillators [(*x*_1_, *y*_1_) and (*x*_2_, *y*_2_)], accounting respectively for the low-voltage fast discharges (i.e., very fast oscillations; Fig. 1*A*, top) and spike-and-wave discharges (Fig. 1*A*, middle). The slowest time scale is responsible for leading the autonomous switch between interictal and ictal states and is driven by a slow-permittivity variable *z* (Fig. 1*A*, bottom). This switching is accompanied by a direct current (DC) shift (Fig. 1*A*, top), which has been recorded *in vitro* and *in vivo* (Ikeda et al., 1999; Vanhatalo et al., 2003; Jirsa et al., 2014).

At some distance before and after seizures (i.e., during the interictal state), the epileptic brain appears to operate “normally” and expresses its rich dynamic repertoire of diverse brain states when driven by noise; these brain states are known as resting-state networks (RSNs; Fox et al., 2005). On fast time scales of 10–500 ms, electrographic recordings identify characteristic oscillatory modes of brain activity showing transient spindle-like behaviors (i.e., fast damped subthreshold oscillations), which repeat themselves intermittently. These waves patterns are strongly dominated by α waves (8–12 Hz; da Silva et al., 1997; Buzsaki, 2006). The existence of fast subthreshold oscillations is a distinguishable feature of systems near a Hopf bifurcation (Fig. 1*D*; Izhikevich, 2007). In Epileptor, a Hopf bifurcation can be configured for *m* = –0.5 and $I_{{\text{ext}}_{2}}=0$ (El Houssaini et al., 2020). However, such parametrization ($I_{{\text{ext}}_{2}}=0$) sets the second population (*x*_2_, *y*_2_) far from its bifurcation, resulting in the loss of interictal spikes when the system is destabilized by noise. To address this problem, we extended Epileptor by another fast time scale of coupled oscillators (*x*_3_, *y*_3_) accounting for transient spindle-like patterns and mathematically equivalent to the normal form of a supercritical Hopf bifurcation (Stefanescu and Jirsa, 2008; Kuznetsov, 2013). This system was already used to retrieve RSNs (Ghosh et al., 2008; Freyer et al., 2011, 2012; Spiegler et al., 2016). Thus, the extended version of Epileptor equations read as follows:

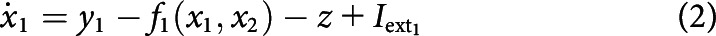


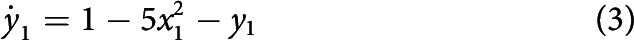


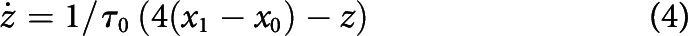


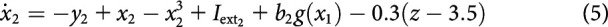


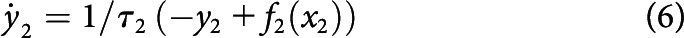


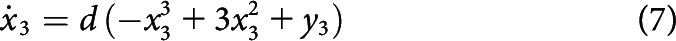


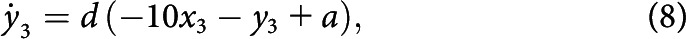
 where

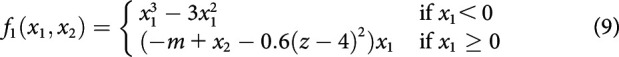


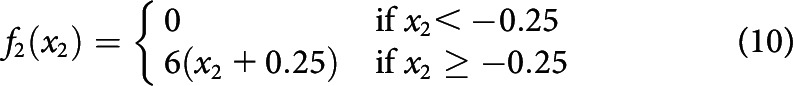


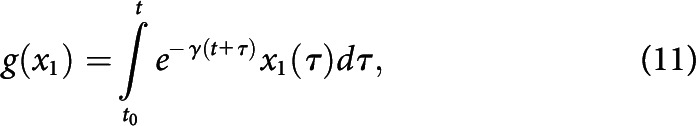
 and *m* = 0, $I_{{\text{ext}}_{1}}=3.1,\tau_{0}=28571,I_{{\text{ext}}_{2}}=0.45,b_{2}=4,\tau_{2}=25$ and γ = 0.01. The characteristic frequency rate *d*, fixed to 0.02, sets the natural frequency of the third subsystem, ∼10 Hz, the most powerful frequency peak observed in electrographic recordings at rest (da Silva et al., 1997; Buzsaki, 2006). The degree of epileptogenicity or excitability of a brain region is represented through the values *x*_0_ and *a*. If *x*_0_ is greater than a critical value (i.e., in supercritical regime), $x_{0,critic}=-2.05$, the brain region can trigger seizures autonomously; otherwise, it is in its equilibrium state (i.e., in subcritical regime). In a similar way, if *a* is greater than a critical value, *a*_critic_ = 1.74, the brain region enters in a stable limit cycle; otherwise, it is in a stable fixed point. We note that in the original Epileptor, τ_0_ = 2857, *b*_2_ = 2, and τ_2_ = 10, the main differences being that IED propagation is shifted toward the interictal period as *g* increases more rapidly and mean spike frequency (the number of IEDs per minute) is decreased to be more realistic. A more detailed description of the original Epileptor model can be found in Jirsa et al. (2014), with an extended bifurcation analysis of its parameters in El Houssaini et al. (2015, 2020). Recent studies link the Epileptor to physiological mechanisms of extracellular potassium accumulation (Chizhov et al., 2018). We coupled the network nodes by permittivity coupling for the Epileptor subpopulation following Proix et al. (2014), and by a fast diffusive coupling for the Hopf subpopulation. The whole-brain network activity is then described by the following equations:

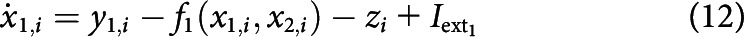


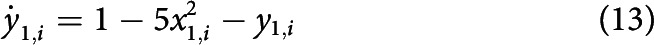


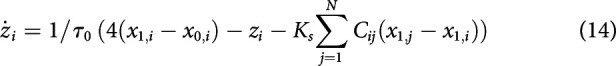


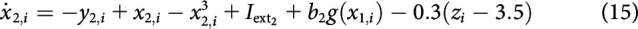


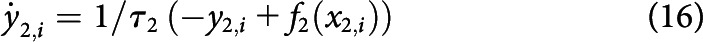


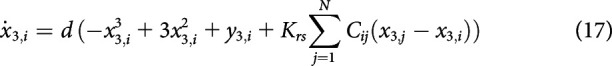


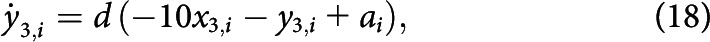
 where *C_ij_* are the weights of the subjects-based SC matrix, and *K_s_* and *K_rs_* are the respective large-scale scaling parameters of the connectivity weights for the Epileptor and Hopf subpopulations. Note that as the BNM of one brain region already takes into account the effect of its internal connectivity, the connection of a region to itself was set to 0 in the connectivity matrix *C_ij_* for the simulations. Also, we assumed the neural transmission via *C_ij_* as instantaneous. Although time delays due to the tract propagation can be of crucial importance for the study of synchronization (Ghosh et al., 2008; Petkoski et al., 2016, 2018; Petkoski and Jirsa, 2019), here being on the phenomenological level, we assumed their impact to be encompassed in the neural masses and we neglected them for the sake of the computational cost. The local bifurcation parameter *a_i_* and the global scaling parameter *K_rs_* are the control parameters with which we studied, by extensive search, to find the optimal dynamical working region of the brain model where the simulations maximally fitted the empirical functional data. The global coupling parameter *K_s_* was fixed to an arbitrary value of 0.1 such that the full system can reproduce seizure (or spike) spread pattern following the clinical criteria of each patient (Table 2). The final parameter values were chosen to fit the extended Epileptor against the experimental data, where the combination of the three ensemble *x*-variables was matched visually against the electrographic signatures of epileptic patient recordings. We found that plotting the following:

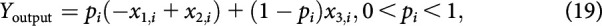
 where the parameter *p*_i_ scales the proportion of the respective activity during the different processes (i.e., ictal and interictal period), as a function of time, bore a striking resemblance with the empirical SEEG signals. From this model, three different case scenarios can then be defined. The first one is the resting state without any epileptiform activity (or spike-free resting state). In this case, the epileptogenic population is silent and *p* is set to 0.1 (Fig. 2*A*). The second and third cases are resting state with interictal spikes (or spiking resting state) and resting state with seizures (or bursting resting state), respectively. Here, the epileptogenic population is dominant and *p* is set closer to 1, or in this case to 0.9 for the epileptogenic zone (EZ)/primary irritative zone (IZ1) and 0.7 for the propagation zone (PZ)/secondary irritative zone (IZ2; Fig. 2*B*,*C*). See Definition of the epileptogenic brain networks subsection for the definition of the different zones. For the purpose of our study, we focused only on the two-first case scenarios.

**Table 2.** Results of EZ/IZ1 and PZ/IZ2 prediction from SEEG signals for each patient

Patient	EZ/IZ1	PZ/IZ2
P1	rLOFC, rTmP	rRMFG, lRMFG
P2	lLOCC	lFuG, lIPC, lSPC
P3	lIns	lPoG
P4	lPCG, lCMFG, lSFG	lPrG, lSPC, lPoG
P5	rPrG	rCMFG
P6	rAmg, rTmP, rLOFC	rFuG, lPHiG, rITG
P7	rAmg, rHi	rITG, rTmP
P8	rLgG, rPHiG	rHi, rFuG, rIPC, rLOCC, rSPC, rITG
P9	rMOFC, rFP, rRMFG, rPOr	rPop, rMTG, rLOFC
P10	rHi, rAmg	rLOFC, rMTG
P11	rHi, rFuG, rEntC, rTmP	lFuG, rITG
P12	rFuG	rEntC, rIPC, rHi
P13	lAmg, lHi, lEntC, lFuG, lTmP, rEntC	lMTG, rMTG, lIns
P14	rLgG, rLOCC, rCun, rPC	rPCunC, lCun, rPHiG

r, right; l, left; Amg, Amygdala; CMFG, Caudal Middle Frontal Gyrus; Cun, Cuneus; EntC, Entorhinal Cortex; FP, Frontal Pole; FuG, Fusiform Gyrus; Hi, Hippocampus; Ins, Insula; IPC, Inferior Parietal Cortex; ITG, Inferior Temporal Gyrus; LgG, Lingual Gyrus; LOCC, Lateral Occipital Cortex; LOFC, Lateral Orbito-Frontal Cortex; MOFC, Medial Orbito-Frontal Cortex; MTG, Middle Temporal Gyrus; PC, Pericalcarine; PCG, Posterior Cingulate Gyrus; PCunC, PreCuneus Cortex; PHiG, ParaHippocampal Gyrus; PoG, PostCentral Gyrus; Pop, Pars Opercularis; POr, Pars Orbitalis; PrG, Precentral Gyrus; RMFG, Rostral Middle Frontal Gyrus; SFG, Superior Frontal Gyrus; SMG, SupraMarginal Gyrus; SPC, Superior Parietal Cortex; STG, Superior Temporal Gyrus; TmP, Temporal Pole.

**Figure 2.**
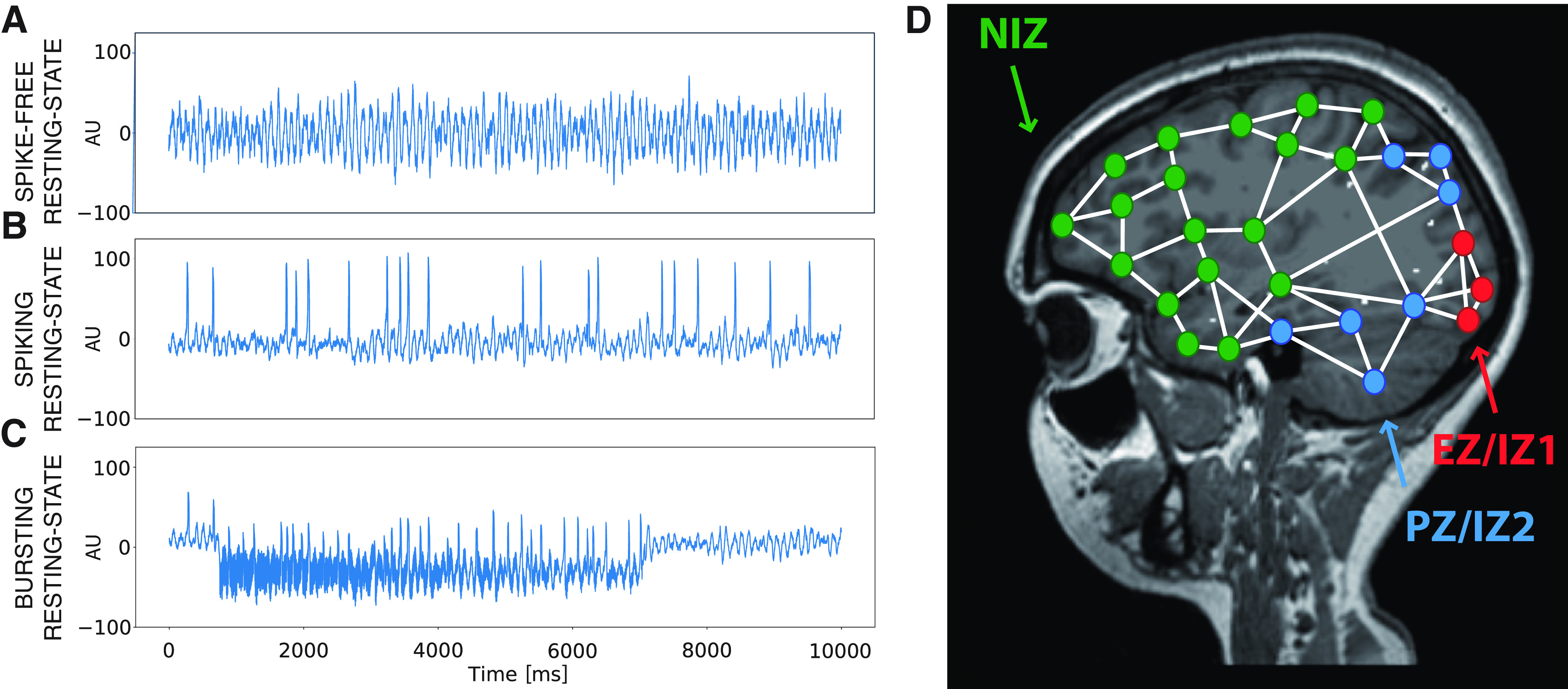
Extended Epileptor dynamics and spatial distribution of epileptogenicity. ***A***–***C***, Time series of the three different case scenarios of the Extended Epileptor model, namely resting state without any epileptiform activity (with *p* = 0.1 in Eq. 19 and *x*_0_ = –2.5 in Eq. 14; ***A***), resting state with interictal spikes (here with *p* = 0.7 and *x*_0_ = –2.07 as example; ***B***), and resting state with seizures (here with *p* = 0.7 and *x*_0_ = –1.9 as example; ***C***). ***D***, When embedded into the network and in the spike-free resting-state scenario (***A***), the regions set in the EZ/IZ1 (red nodes) and PZ/IZ2 (blue nodes) have a very low excitability value *x*_0,_*_i_* ($x_{0,i}\leq x_{0,critic}-0.5$) and high value *a_i_* (*a_i_* > *a_critic_*), and all other regions set in the NIZ (green nodes) have the same excitability value *x*_0,_*_i_* but an elevated excitability value *a_i_* (*a_i_* = *a_critic_*); in the spiking resting-state scenario, the regions in the EZ/IZ1 have a low *x*_0,_*_i_* ($x_{0,i}\leq x_{0,critic}$) and high *a_i_* (*a_i_* > *a_critic_*), the regions in PZ/IZ2 have lower *x*_0,_*_i_* ($x_{0,i}\leq x_{0,critic}-0.2$) and high *a_i_* (*a_i_* > *a_critic_*), and all other regions in the NIZ have a very low *x*_0,_*_i_* ($x_{0,i}\leq x_{0,critic}-0.5$) and elevated *a_i_* (*a_i_* = *a_critic_*; ***B***). The white links represent the anatomical structural connections. See Definition of the epileptogenic brain networks for the description of the different zones.

#### Numerical implementation

All the simulations were performed with TVB using a stochastic Heun's integration scheme (Mannella, 2002) with a time step of 0.1 ms, a simulation time of 20 min, and random initial conditions drawn from a normal distribution (N(0,1)). Additive white Gaussian noise was introduced in the state variables *x*_2_ and *y*_2_, with a mean of 0 and noise strength of 0.00025, as well as in *x*_3_ with a mean of 0 and noise strength of 0.02. The data corresponding to the first 20 s were always discarded from the analysis to avoid initial transient dynamics.

To test the emergence of ultra-slow fluctuations, we estimated the BOLD signal changes associated with the simulated neural activity (Eq. 19) using the Ballon–Windkessel hemodynamic model (Friston et al., 2003) implemented in TVB. The Ballon–Windkessel model describes the coupling of perfusion to the BOLD signal, with a dynamical model of the transduction of neural activity into perfusion changes. The simulated BOLD signal was downsampled at 3600 ms to match the time resolution (TR) of the empirical fMRI signals. The global mean signal was then regressed out from each region's time series, and temporal bandpass filtering was performed to retain frequencies between 0.01 and 0.1 Hz using a third-order Butterworth filter to reproduce empirical conditions.

#### Definition of the epileptogenic brain networks

Epileptogenic brain networks were evaluated by two different methods. The first one consisted of the visual inspection and interpretation by the expert epileptologist (F.B.) of the different measurement modalities gathered throughout the two-step procedure (noninvasive and invasive) of the comprehensive presurgical evaluation of each patient. The second method consisted in the application of signal-processing techniques on the invasive SEEG measurements, which have been used in previous studies (Bartolomei et al., 2013b, 2017). In particular, SEEG signals were used to refine the zones involved by different epileptogenic processes (Bettus et al., 2011), as follows: the EZ/IZ1, the PZ/IZ2, and the noninvolved zone (NIZ) are determined for each patient. EZ/IZ1 is defined as the subset of brain regions involved in the generation of seizures that may also exhibit IEDs. PZ/IZ2 is defined as those regions only secondarily involved in seizures and that produce interictal spikes. Finally, NIZ is defined as structures without epileptiform discharges during clinical monitoring. The identified zones for each patient are provided in Table 2.

For the simulations, the different regions set respectively in EZ/IZ1 and PZ/IZ2 were used to reconstruct the epileptogenic networks in our individual virtual epileptic brains. We defined a spatial distribution map of epileptogenicity or excitability where each brain region, *i*, was characterized by an excitability value, *x*_0,_*_i_*, which quantifies the ability of the Epileptor subpopulation to trigger an epileptogenic discharge or not; and an excitability value *a_i_*, which quantifies the ability of the Hopf subpopulation to generate self-sustained oscillations. The spatial map of epileptogenicity comprises the excitability values of the EZ/IZ1, PZ/IZ2, and all other NIZ regions (Fig. 2*D*).

#### Resting-state functional connectivity analysis

**Figure 3.**
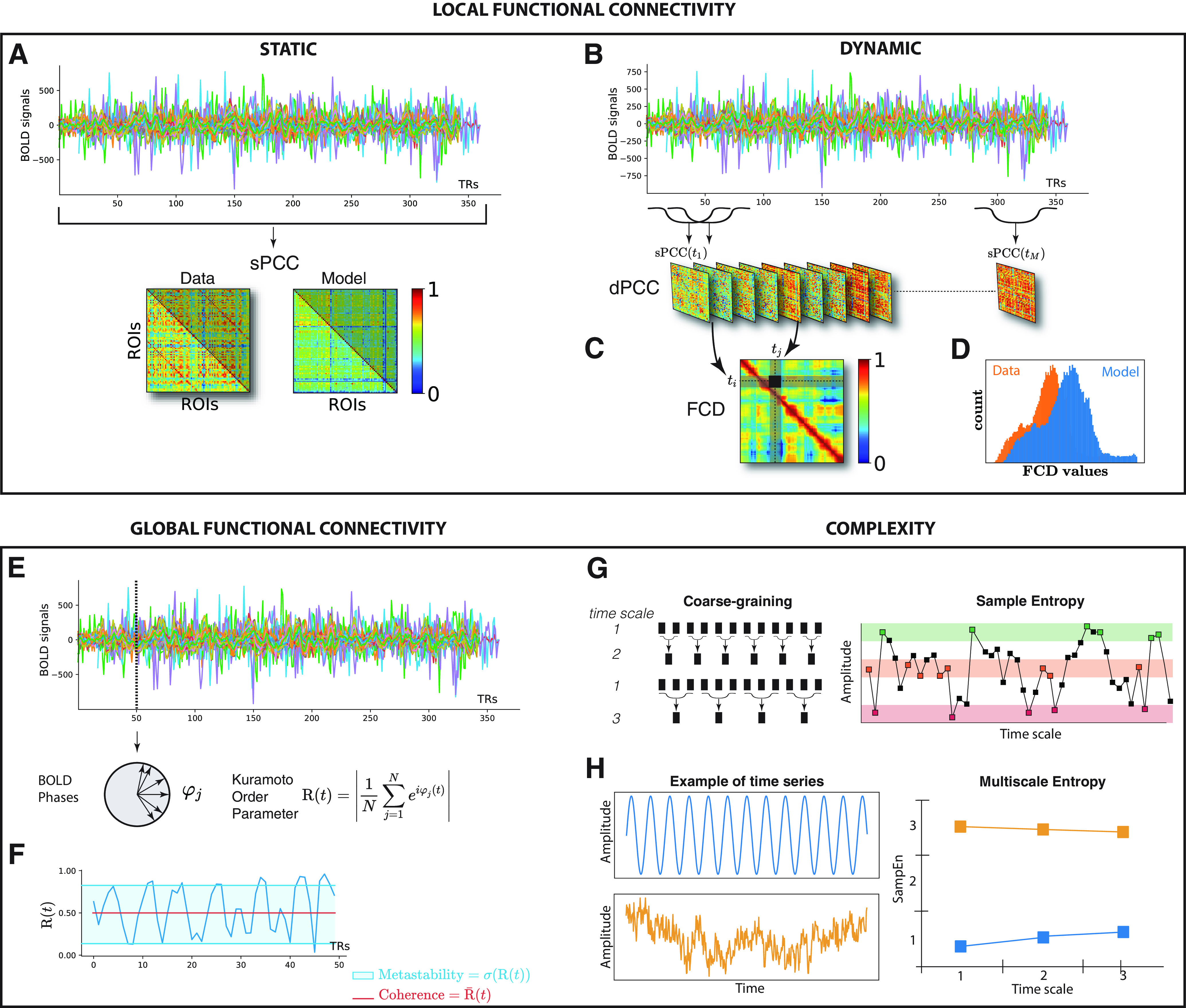
Methods for measuring and fitting RS-FC. ***A***, sPCC is estimated as the Pearson correlation coefficients between each pair of brain regions over the whole time window. The fitting of the sPCC is measured by the Pearson correlation coefficient between corresponding elements of the upper triangular part of the matrices. ***B***, dPCC is estimated as the series of sPCC matrices from the windowed segment. ***C***, FCD is estimated as the Pearson correlation coefficient between each pair of sPCC matrices. ***D***, For comparing the FCD statistics, the upper triangular elements of the matrices were collected, and the simulated and empirical distribution were compared by means of the Kolmogorov–Smirnov distance between them. ***E***, The phase synchronization of the BOLD signals is measured by the Kuramoto order parameter *R*. ***F***, The coherence and the metastability are estimated as the mean $\bar{R}$ and the standard deviation σ*_R_* of the Kuramoto order parameter across time. The phase synchrony is fitted by minimizing the absolute value of the difference between the empirical and simulated coherence and metastability. ***G***, Schematic illustration of the coarse-graining (left) and an exemplary time series is shown to illustrate the procedure for calculating sample entropy for the case *m* = 2 and a given positive real value *r* (denoted by the height of the colored bands; right). ***H***, The MSE profiles are fitted by minimizing their RMSD.

FC describes brain function through estimates of the covariant links between two signals (originating from different brain regions), reflecting how different brain areas coordinate their activities. Functional connections were explored from both local (i.e., at the regional level) and global (i.e., at the whole-brain level) perspectives using a set of four measures of their spatiotemporal dynamics that have been widely used to estimate FC from RS-fMRI signals (for review, see Smitha et al., 2017). An additional metric was used to explore the complexity of the fMRI signals. Figure 3 shows a summary of these metrics. The simulated and empirical data are analyzed using TVB analysis tools.

#### Local functional connectivity

##### Static PCC.

The first metric applied is the most classic and widely used to infer the strength of functional connections, namely the Pearson correlation coefficients (PCCs). The PCC consists of estimating the (linear) temporal correlations between each pair of brain regions over the whole time window acquisition [we talk about “static” PCC (sPCC); Bandettini et al., 1993; Biswal et al., 1995]; that is, for each pair of regions *i* and *j*, the corresponding time series *x_i_*(*t*) and *x_j_*(*t*) of size *N* are used to calculate the correlation coefficients *r_ij_* (Fig. 3*A*), as follows:

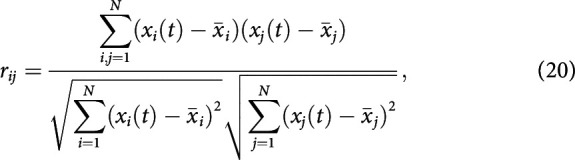
 where the notation $\bar{\mathrm{...}}$ denotes the mathematical mean operator. This sPCC was estimated in the same way from the empirical and simulated fMRI BOLD signals. Since the matrices are symmetric, we compared the modeled and empirical sPCC matrices by using the Pearson correlation coefficient between the corresponding elements of their upper triangular part.

##### Dynamic PCC.

To consider the temporal dynamics of RS-FC, we computed the FC using a sliding temporal window approach (Hutchison et al., 2013; Allen et al., 2014; Hansen et al., 2015). In this approach, each full-length BOLD signals of 20 min (either empirical or simulated) was split up into a mean of 328 sliding windows of 60 s, overlapping by56.4 s (i.e., an increment of 1 TR). For each sliding window centered in time *t*, we calculated a separate sPCC matrix [sPCC(t)]. This procedure resulted in a series of sPCC matrices that describe the time-resolved behavior of connectivity over the entire duration of the fMRI BOLD signals, namely the dynamic PCC (dPCC; Fig. 3*B*). Subsequently, to study the evolution of the dPCC over time with reduced dimensionality, we computed a time-versus-time matrix representing the functional connectivity dynamic (FCD; Hansen et al., 2015), where each entry (*t_i_*, *t_j_*) was defined by the Pearson correlation coefficient between the upper triangular parts of the two matrices $\text{sPCC}(t_{i})$ and $\text{sPCC}(t_{j})$ centered at time *t_i_* and *t_j_*, respectively (Fig. 3*C*).

For comparing the FCD statistics, we collected the upper triangular elements of the matrices and generated their cumulative distribution function. Then, we compared them through the Kolmogorov–Smirnov (K–S) distance, which quantifies the maximal difference between the two samples (Fig. 3*D*; Deco et al., 2017a).

#### Global functional connectivity

The global network dynamics were quantified using coherence and metastability, which are defined by the mean and the standard deviation of synchronization over time (Shanahan, 2010; Wildie and Shanahan, 2012; Cabral et al., 2014). The phase synchrony of the empirical and simulated network dynamics was evaluated using the Kuramoto order parameter *R*(*t*) (Kuramoto, 1984), which is defined as follows (Fig. 3*E*):

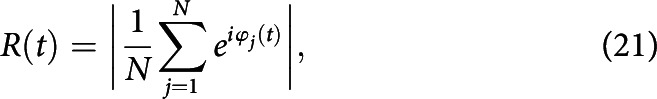
 where *N* is the number of brain regions in the network and $\varphi_{j}(t)$ is the instantaneous phase of each region *j* estimated using Hilbert transform (Glerean et al., 2012; Ponce-Alvarez et al., 2015). *R* measures the phase uniformity and varies between 0 for a network in a fully desynchronized—or incoherent—state and 1 for full synchronization. Thus, for calculating the coherence $\bar{R}$ and metastability σ*_R_* of the empirical and simulated BOLD signals, we computed the instantaneous phase $\varphi_{j}(t)$ of the signal of each region *j* using the Hilbert transform. The Hilbert transform yields the associated analytical signals *s*(*t*), representing a signal in the time domain as a rotating vector with an instantaneous phase, $\varphi_{j}(t)$, and an instantaneous amplitude, *A*(*t*) [i.e., $s(t)=A(t)\cos(\varphi_{j}(t))$]. The phase and amplitude are given by the argument and the modulus, respectively, of the complex signal *z*(*t*), given by z(t)=s(t) + iH(s(t)), where *i* is the imaginary unit and H(s(t)) is the Hilbert transform of *s*(*t*).The simulated coherence and metastability were compared with the empirical values of synchronization between the different brain regions across time by using as a measure of similarity the absolute value of the difference (Wildie and Shanahan, 2012; Deco et al., 2017b; Lee and Frangou, 2017).

#### Complexity analysis: multiscale entropy

Introduced as a measure of complexity of physiological signals, multiscale entropy (MSE; Costa et al., 2002, 2005) is thought to evaluate the presence of long-range correlations in the behavior under scrutiny by taking into consideration the multiple time scales on which the overall dynamics operate. Technically, MSE consists in computing sample entropy (Richman and Moorman, 2000) of the successively coarse-grained signals, namely downsampled versions of the original signal at different (time) scales. For a detailed description of MSE measure and its relevance for the analysis of signal complexity see Courtiol et al. (2016).

Briefly, a time series $x=\{x_{1}...x_{N}\}$ of size *N* is divided into segments that correspond to consecutive nonoverlapping time intervals of length τ_SF_, where τ*_SF_* represents the scale factor and takes integer values ≥1. All data points in each segment are replaced by the average value of that segment, thus producing a new time series, $y^{\tau_{\text{SF}}}=\{y_{j}^{\tau_{\text{SF}}},j=1\cdots N/\tau_{\text{SF}}\}$, called a coarse-grained time series, the length of which is equal to that of the original, *N*, divided by the scale factor τ_SF_ (Fig. 3*G*), as follows:

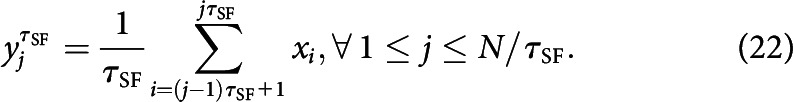


For scale 1, *y*^1^ is simply the original time series. Then, the sample entropy (SampEn), quantifying the predictability or regularity of a time series (Richman and Moorman, 2000), is applied for each time series $y^{\tau_{\text{SF}}}$. It is defined as the negative natural logarithm of the conditional probability that two similar sequences of *m* consecutive data points in a time series of length N′ within a given tolerance *r* normalized to the standard deviation of the time series, will remain similar when the next point *m* + 1 is also included in the sequence (Fig. 3*G*). Then, for each time scale τ*_SF_*:

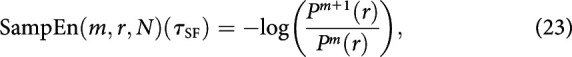
 where the quantity $P^{m+1}(r)/P^{m}(r)$ is the conditional probability described above. Note that BOLD time series usually comprise few data points, and the coarse-grained procedure in MSE with large scale factor may result in short data length and, subsequently, in unreliable SampEn estimation. Prior studies suggested that data length of 10*^m^* to 20*^m^* should be sufficient for a robust calculation (Richman and Moorman, 2000). Therefore, following several studies using MSE in fMRI recordings, we set *m* to 1 and *r* to 0.35 (Nakagawa et al., 2013; Yang et al., 2013) and computed MSE over scales 1:13 (3.6–46.8 s). The empirical and simulated MSE profiles were compared using the root mean square distance (RMSD; Fig. 3*H*).

#### Global similarity

Based on the previously described five fitting metrics and having the considerations that (1) absolute value of the difference is better as it gets closer to 0, (2) the Pearson correlation coefficient is better as it gets closer to 1, (3) K–S is better as it gets closer to 0, and (4) RMSD is better as it gets closer to 0, we developed an additional fitting metric, the global similarity (GS), to express all these conditions in a single numerical value (Kehoe et al., 2017). MSE is the only metric that portrays the complexity, hence we gave more importance to RMSD in the expression of GS. Thus, GS depends quadratically on RMSD, while it depends only linearly on the other metrics, as follows:

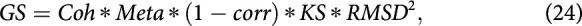
 where *Coh* and *Meta* represent the absolute value of the difference between the empirical and simulated coherence and metastability, respectively; *corr* is the Pearson correlation coefficient between the empirical and simulated sPCC matrices, and *KS* is the K–S distance between the empirical and simulated FCD histograms. Then, we used GS to find the optimal working region in an automated way by defining it as the values of the free model parameters, namely the local bifurcation parameter *a* and the global coupling strength *K_rs_*, where GS exhibited its minimal value. Note that the values of GS were normalized (between [1,2]).

#### Statistical analysis

When comparing two SC or RS-FC distributions from a particular measure of connectivity, we performed a two-tailed Wilcoxon rank-sum test, testing the null hypothesis that there existed no difference between the calculated measures at both distributions. The statistical significance level was set to *p* < 0.05, and so the *z* score was greater than |1.96|.

## Results

The main goal of our study was to identify network mechanisms behind the RS-FC patterns observed in epileptic patients, and to characterize how epileptogenic brain regions express themselves outside the seizure (i.e., during the interictal state). To this end, we developed a novel large-scale BNM linking the underlying anatomical SC of each patient (derived from MRI scans) with the local functional dynamics of each brain area to emulate the characteristics of spontaneous whole-brain dynamics, as observed in functional neuroimaging data.

The spatiotemporal structures of spontaneous fMRI BOLD fluctuations were characterized using the following five RS-FC estimates: (1) sPCC and (2) dPCC (or FCD; the static PCC describes the mean spatial structure of the resting-state activity, whereas the dynamic PCC captures the temporal structure of those spatial correlations); (3) coherence and (4) metastability, which quantify the level of phase synchronization between brain regions across time; and (5) MSE, which measures the level of complexity within each brain region across multiple temporal scales.

### Structural reorganization in epileptic patients not captured by SC weights

The SC from the brains of epileptic patients and healthy control subjects were compared using two typical graph theory measures, namely node strength and streamline counts. These metrics can provide good predictions of the optimal working point of the brain model, as SC weights appear in the brain model equations as a linear combination with the remote brain region activity. The mean values of the streamline counts and mean structural strength of the nodes did not differ significantly between the two groups, and also between patients with MRI-positive and MRI-negative results. However, the small sample size of the healthy group limits the statistical power of this analysis.

### Shift of the optimal working point of brain model toward lower excitability in patients with epilepsy

**Figure 4.**
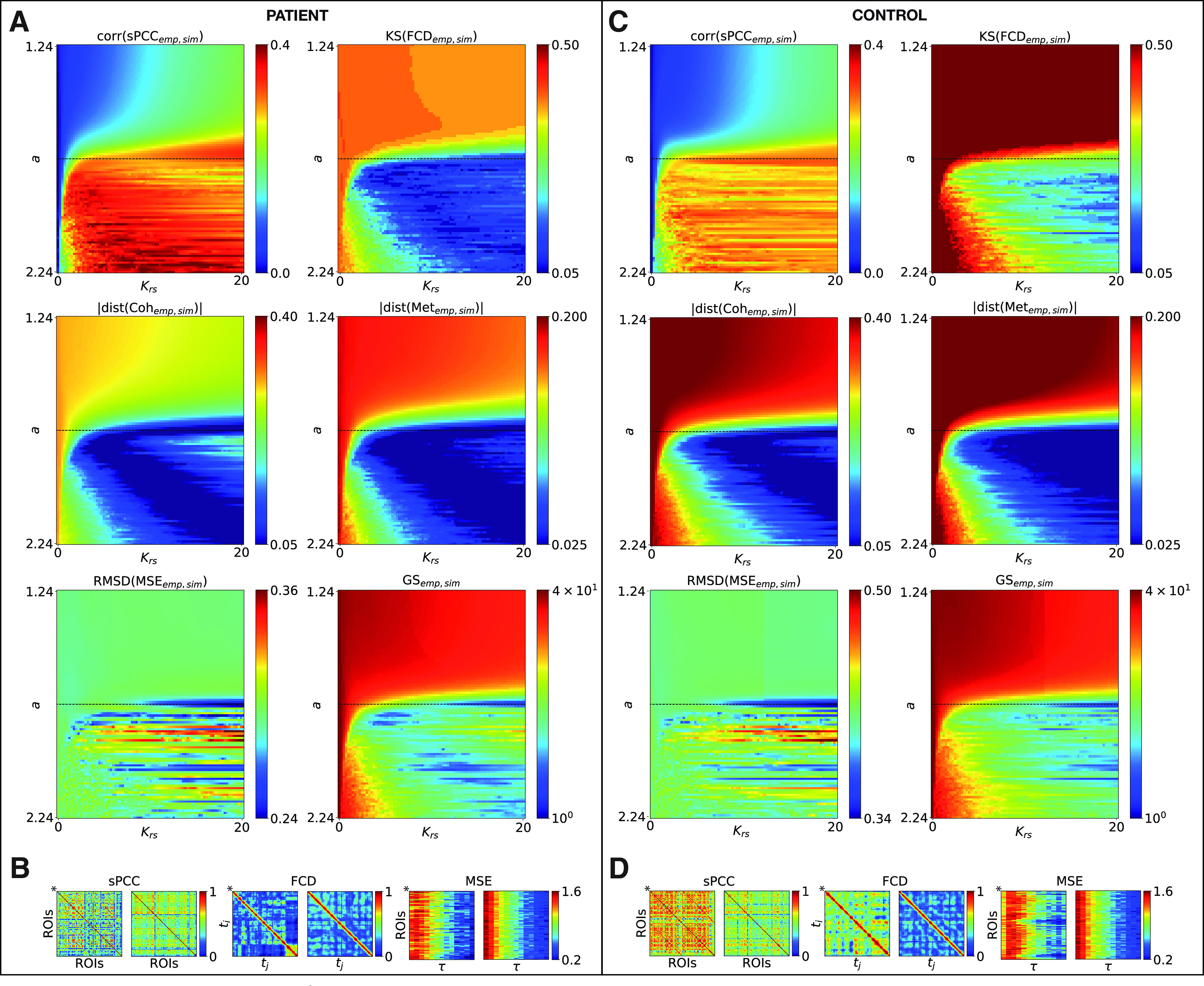
Whole-brain parameter space explorations and RS-FC fitting for an exemplary epileptic patient and healthy control subject. ***A–C***, The six metrics for assessing the RS-FC fitting between the simulated and empirical data are shown color coded as a function of the global coupling *K_rs_* (*x*-axis) and the bifurcation parameter *a* (*y*-axis). Top row, Correlation coefficient between the empirical and simulated sPCC matrices (left) and the K–S distance between the empirical and simulated FCD histograms (right). Middle row, Absolute value of the difference between the empirical and simulated coherence (left) and metastability (right). Bottom row, RMSD between the empirical and simulated MSE matrices (left) and GS metric (right). The horizontal black dashed lines represent the critical point. ***B–D***, Simulated sPCC, FCD, and MSE matrices for the optimal working point. For comparison, the same matrices are plotted for the empirical data (denoted by*).

We first investigated the dynamical properties of the optimal working point of the brain model that was able to fit the characteristics of the empirical fMRI data from 14 epileptic patients and 5 healthy control subjects. fMRI BOLD activity was simulated with the extended Epileptor model in the case of absence of epileptiform discharges (i.e., in the spike-free resting-state scenario with *p* = 0.1 for all brain regions in Eq. 19) with the brain regions coupled through the empirical subject-specific SC matrix *C_ij_* as extracted from the DTI-based tractography (normalized to unity). Using our model, we studied whether the patients' SC led to altered RS-FC at the dynamical level, and, particularly, how this depended on the two free parameters of the model, namely, the local bifurcation parameter *a_i_*, that was homogeneously modified over all regions (i.e., *a_i_* = *a*, for all *i*), and the global coupling *K_rs_*. We performed a parameter space exploration of these two parameters and characterized the whole-brain RS-FC of each subject. For this, we used the previously introduced metrics static and dynamic PCC, coherence, metastability, and MSE for each set of parameters (*a*, *K_rs_*). In this paragraph, we additionally used the GS to refine the working point. These six metrics characterize computationally the bifurcation properties of the full network dynamics. Then, we computed the best fit between the simulated and empirical RS-FC-based metric values. All five parameter spaces presented in Figure 4*A–C* display a full picture of the spatiotemporal organization of the system.

**Figure 5.**
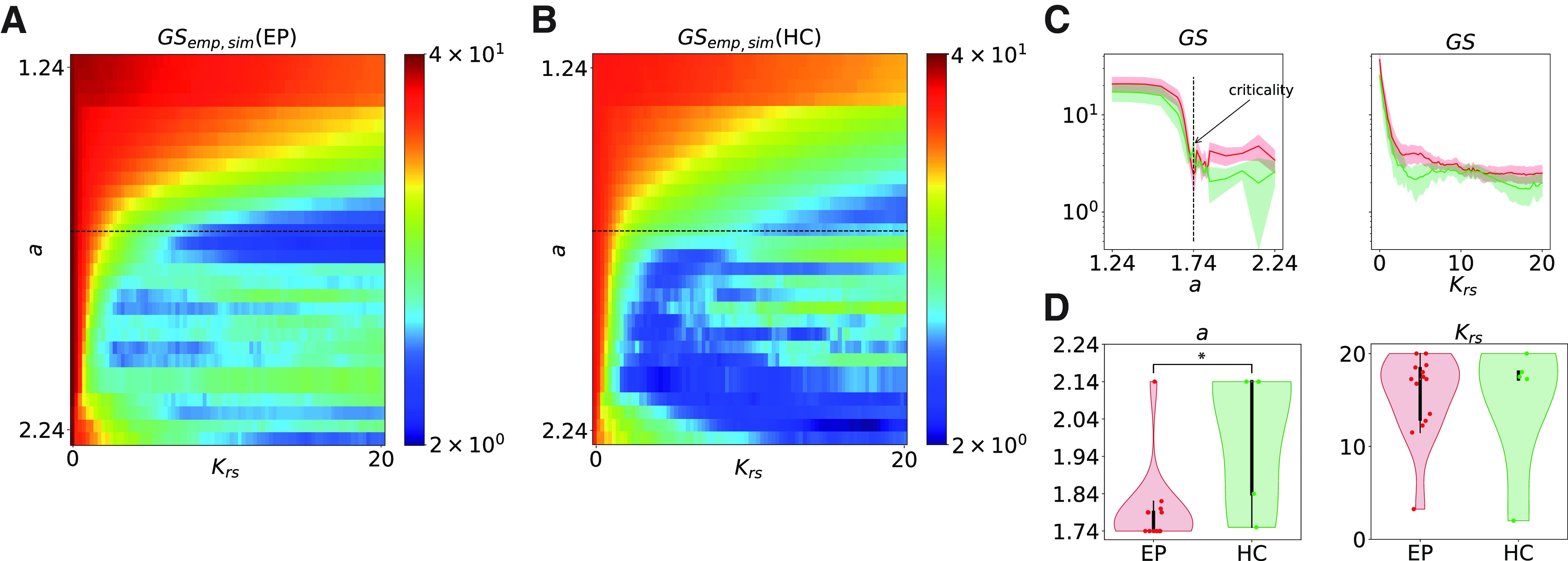
Group comparison of the optimal working point in the brain models. Median group results. ***A***, ***B***, Level of fitting of the GS between the simulated and empirical data are shown color coded as a function of the bifurcation parameter *a* (*y*-axis) and the global coupling *K_rs_* (*x*-axis) for the epileptic group (EP; ***A***) and the healthy group (HC; ***B***). The horizontal black dashed lines represent the critical point. ***C***, Minimal level of fitting of the GS as a function of the local bifurcation parameter values *a* (left) and coupling strength *K_rs_* (right), for the epileptic group (red curves) and control group (green curves). Areas of faded colors represent the standard error intervals. ***D***, Distribution of optimal local bifurcation parameter values *a* (left) and coupling parameter values *K_rs_* (right), for EPs (red violin plot) and HCs (green violin plot). *p = 0.038.

Figure 4*A* shows the results of this analysis for an exemplary epileptic patient (P1). As can be observed in Figure 4*A* (top row, left panel), the correlation between the empirical and simulated sPCC matrices is sensitive to the large-scale coupling strength *K_rs_* and the bifurcation parameter *a*. The model shows the best agreement with empirical data for a broad range of parameters (Fig. 4*A*, area indicated with hot colors); indeed, a large region of model parameters is consistent with the empirical data and reaches a correlation of up to 0.40 between empirical and simulated sPCC matrices. This is within the values of similarity reported in previous studies that have simulated subject-specific brain dynamics at the large-scale level (see for example Deco et al., 2013). In Figure 4*A* (right), the best fitting of the spatiotemporal characteristics of the empirical RS-fMRI data can be found at the minimum of the K–S distance between the empirical and simulated FCD histograms for a smaller range of parameters (Fig. 4*A*, area indicated with cold colors). In Figure 4*A* (middle row), the phase synchronization behavior of the system obtained from the simulations is compared with the empirical fMRI BOLD data of the patient for the same range of model parameters. In the range of best agreement with FCD (Fig. 4*A*, cold colors), the absolute difference between the empirical and simulated coherence (Fig. 4*A*, left), and metastability (Fig. 4*A*, right), respectively, is minimized (Fig. 4*A*, area also indicated with cold colors). Note that the calculated coherence (or degree of phase synchrony) is moderate ($0.3<\bar{R}<0.4$), indicating that the system is neither fully synchronized ($\bar{R}$ close to 1) nor incoherent ($\bar{R}$ close to 0), and the coherence exhibits maximal variability or metastability ($0.2<\sigma_{R}<0.3$). Moreover in the last row of Figure 4*A*, the RMSD between the empirical and simulated MSE matrices displays the best agreement for a limited range of parameters close to the Hopf bifurcation (Fig. 4*A*, horizontal black dashed line). In Figure 4*A* (right), the GS is plotted to refine the best-fit zone between all metrics (area indicated with cold colors). All other patients exhibit RS-FC configuration patterns that were highly similar to those of patient P1 (Fig. 5*A*, median group results of GS).

Subsequently, the same analysis was performed on the simulated data obtained from the healthy control subjects. The results of this analysis are presented in Figure 4*C* for an exemplary healthy subject. The plots show that the data obtained with the model indicate an optimal working point into a similar range of parameters compared with patient P1. All other subjects have very similar fitting patterns (Fig. 5*B*, median group results of GS), although the best agreement with the data is obtained for a larger range of parameters, in particular in the supercritical regime (i.e., self-sustained oscillations).

Furthermore, we compared the dynamic working point of the two groups with regard to the GS parameter space in Figure 5*C*. The GS between the empirical and modeled data is more sensitive to the local bifurcation parameter *a* (Fig. 5*C*, left) than the global coupling strength *K_rs_* (Fig. 5*C*, right) for both groups. In particular, for the epileptic patients, the optimal working point is obtained when the brain regions operate in the vicinity of the supercritical Hopf bifurcation (i.e., close to a≃1.74; Fig. 5*D*, left). For the healthy subjects, the best fit is obtained in the supercritical regime (Fig. 5*D*, left). However, there is a quite large region with a U-shaped dependence of *K_rs_* on *a*, where there is relatively good fitting (Fig. 5*B*). This could suggest either that there are several working points for the healthy brain or that it could be a result of the small number of subjects. The dynamic working point shifts significantly (*p* = 0.038) to lower bifurcation parameter values in epilepsy compared with the healthy state, where the system remains in the oscillatory region. The simulated data obtained at the optimal working point for the exemplary epileptic patient P1 (i.e., at *a* = 1.74 and *K_rs_* = 20) and the healthy control subject (i.e., at *a* = 1.74 and *K_rs_* = 20) are shown in Figure 4*B–D*. For comparison, the same matrices are plotted for the empirical data.

Note that due to the lack of simultaneous physiological recording during the RS-fMRI acquisition, no additional physiological noise modeling has been applied and some aliasing artifacts (e.g., cardiac and respiratory rhythms) could be still present in the data. However, regressing them out also removes a meaningful part of the brain signal. Indeed, we performed an ICA-based noise removal for all of our subjects and the best-fit GS decreases by ∼54% for the epileptic group and by ∼70% for the control group, although the landscape and working region of the model remain topologically identical and quantitatively similar. Thus, this preprocessing procedure does not make a qualitative change in the results. The denoising of the RS-fMRI is still being debated in the field (Bright and Murphy, 2015) and more in-depth investigation of this issue is beyond the scope of our study here, which has a modeling focus.

### IEDs do not change connectivity patterns

The impact of the presence of IEDs in brain signals on RS-FC features was systematically evaluated by simulating the spontaneous activity with the extended Epileptor in the spiking resting-state scenario (where the parameter *p* was set according to the relative contribution of healthy and interictal activity in Eq. 19). The local bifurcation parameter *a_i_* was fixed for all the regions at the value *a_i_* = *a* = 1.74, where the optimal similarity was observed between the model and the data in a large majority of patients, while the global coupling *K_rs_* was set at the patient-specific optimal value.

**Figure 6.**
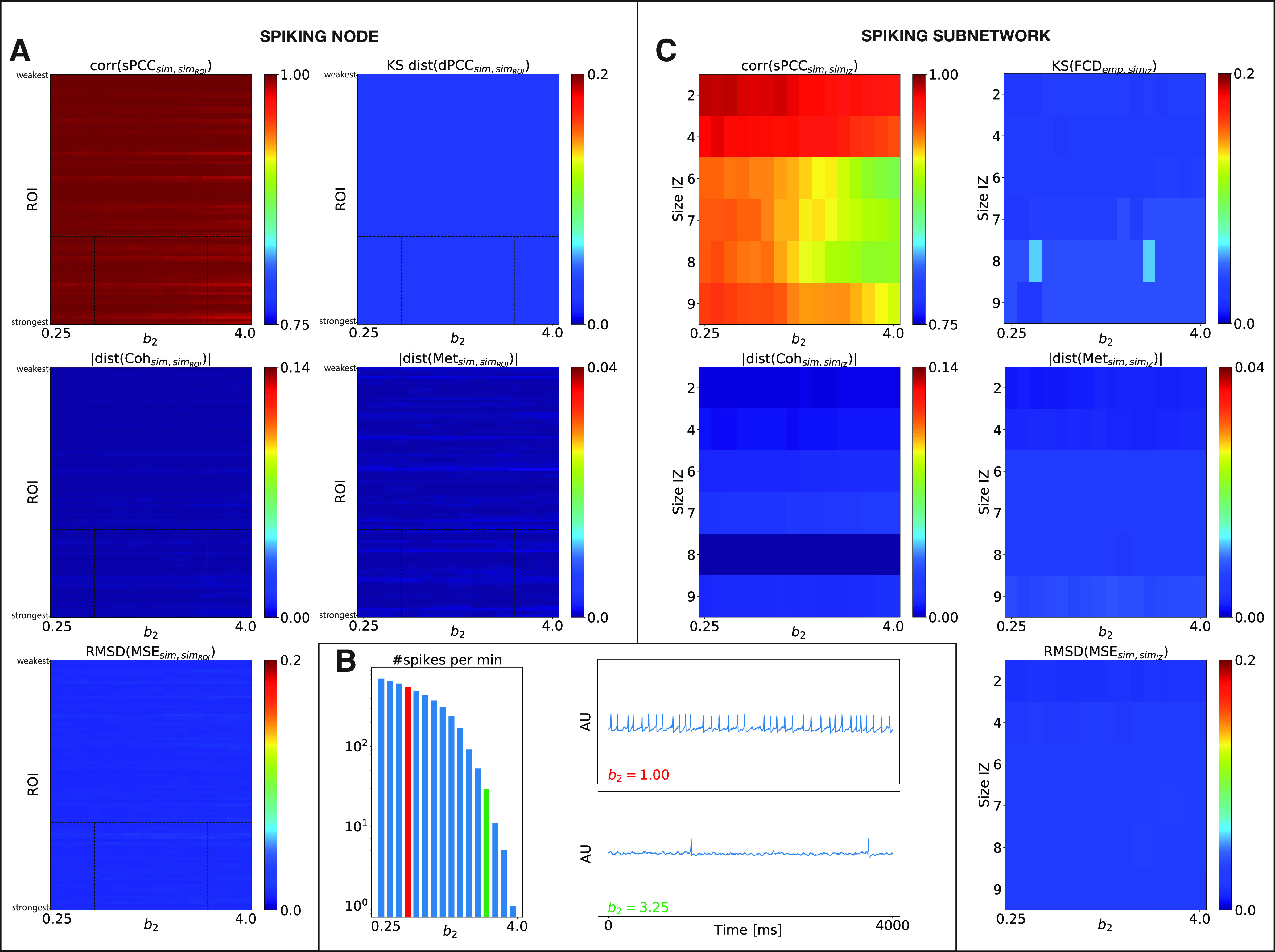
Impact of interictal spikes on the whole-brain RS-FC. ***A***, ***C***, The five metrics for assessing changes between simulated data with and without IEDs in brain signals are shown color coded as a function of spike frequencies linked to the parameter *b*_2_ (*x*-axis) and spiking region ROI sorted by structural node strength (***A***) and the size of the patient-specific IZ (*y*-axis; ***C***). Top row, Correlation coefficient between the two simulated sPCC matrices (left) and the K–S distance between the two simulated FCD histograms (right). Middle row, Absolute value of the difference between the two simulated coherence (left) and metastability (right). Bottom row, RMSD between the two simulated MSE profiles. ***B***, Left, Number of spikes (in minutes) in the mid-connected brain region and (right) its respective neural activity time series for two different values of the parameter *b*_2_ (black dashed lines in ***A***).

**Figure 7.**
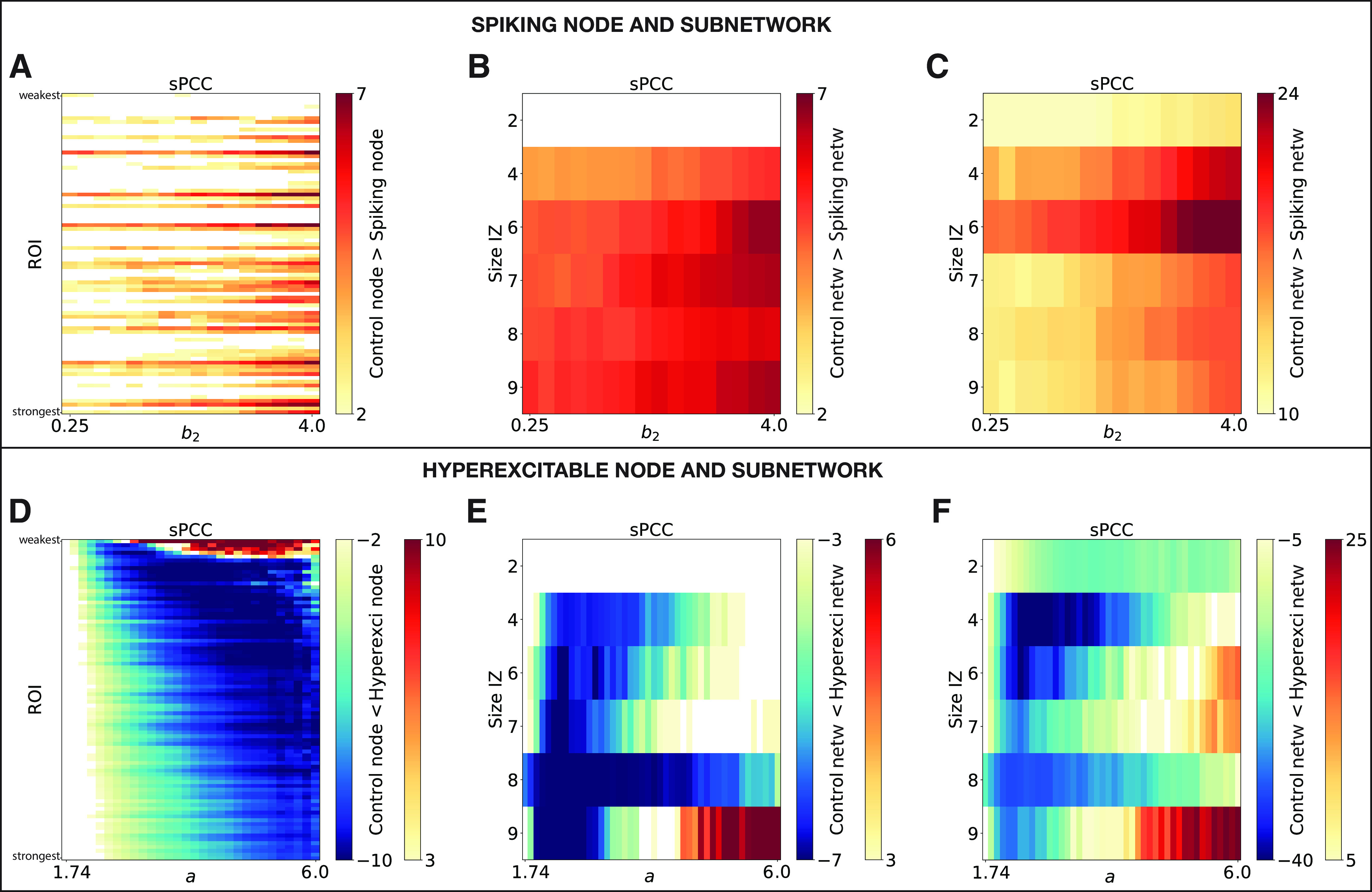
Statistical comparison of the sPCC matrices between the spiking or hyperexcitable case to the control scenario. The *z*-score values are color coded as a function of spike frequency *b*_2_ (top row) or bifurcation parameter *a* (*x*-axis; bottom row), and spiking or hyperexcitable region ROI sorted by structural node strength (left column) or the size of the patient-specific IZ (*y*-axis; middle and right columns). ***A–F***, Comparisons are for spiking or hyperexcitable region with the rest of the brain (***A–D***), within IZ (***B–E***), and IZ with the rest of the brain (***C–F***).

We first introduced one spiking region in the BNM and we investigated whether the spike frequency, modulated by the parameter *b*_2_ in Equation 15, led to changes in RS-FC at the whole-brain dynamical level and, in particular, how this depended on the structural strength of that region. Parameter space exploration was performed by successively modifying the selected spiking region [called the region of interest (ROI)] in the SC that produced IEDs, and by varying the parameter *b*_2_. For each set of parameters (ROI, *b*_2_), we calculated the changes in whole-brain RS-FC compared with a control case scenario (i.e., without interictal spikes or in spike-free resting state) using the metrics defined above. The results are shown in Figure 6, where the BNM was computed from the connectome of P1. Figure 6*A* shows that the RS-FC patterns are very slightly affected by the presence and the frequency of interictal spikes, which is increased for lower *b*_2_, as illustrated in Figure 6*B*, and by the structural node strength of the region. Indeed, all the parameter spaces are fully homogeneous. We then performed a deeper analysis by comparing the sPCC connectivity of the selected spiking region with the rest of the brain to the control scenario. The comparison was done by applying a two-tailed Wilcoxon rank-sum test (Fig. 7*A*), which reveals a widespread significant decrease in the connectivity (*p* = 0.04). This means higher connectivity in the control case, as the frequency of interictal spikes decreases for higher *b*_2_ (i.e., when the frequency of spikes becomes comparable to the sampling frequency of BOLD signals).

Furthermore, we analyzed the impact of the presence of a spiking subnetwork on the RS-FC, by simulating individual epileptic brain models with an IZ. The epileptogenicity *x*_0,_*_i_* of the regions belonging respectively to IZ1, IZ2, and NIZ was set according to clinical criteria (Table 2). The spatial distribution of epileptogenicity values is thus heterogeneous across the brain network (Fig. 2*D*), with a small value *x*_0_ for regions in IZ1 ($x_{0,i}<x_{0,\text{critic}}$), smaller excitability for regions in IZ2 ($x_{0,i}\leq x_{0,\text{critic}}-0.2$), and very low excitability for the other regions ($x_{0,i}-0.5\leq x_{0,\text{critic}}$). The BOLD signal was then derived from the neural activity (Eq. 19) using the Ballon-Windkessel model, on which we calculated the RS-FC metrics. The combined results for all the patients are presented in Figure 6*C*. Although increasing the size of IZ increases the impact of spikes, changes are very small for all the metrics. Notably, higher spike frequencies seem to be completely irrelevant for low-frequency BOLD RS-FC. Subsequently, we compared sPCC connectivity of patient-specific IZ with the rest of the brain (Fig. 7*C*), and within IZ (Fig. 7*B*) to the control case scenario (i.e., no spiking subnetwork). Figure 7*B* and *C*, shows a significant decrease in connectivity in the spiking scenario (*p* = 0.00012 and $p=2.5e^{-16}$, respectively), albeit increasing the size of the IZ increases the impact of spikes. Note that for the case of two regions in the IZ (Fig. 7*B*), statistical comparison cannot be performed.

**Figure 8.**
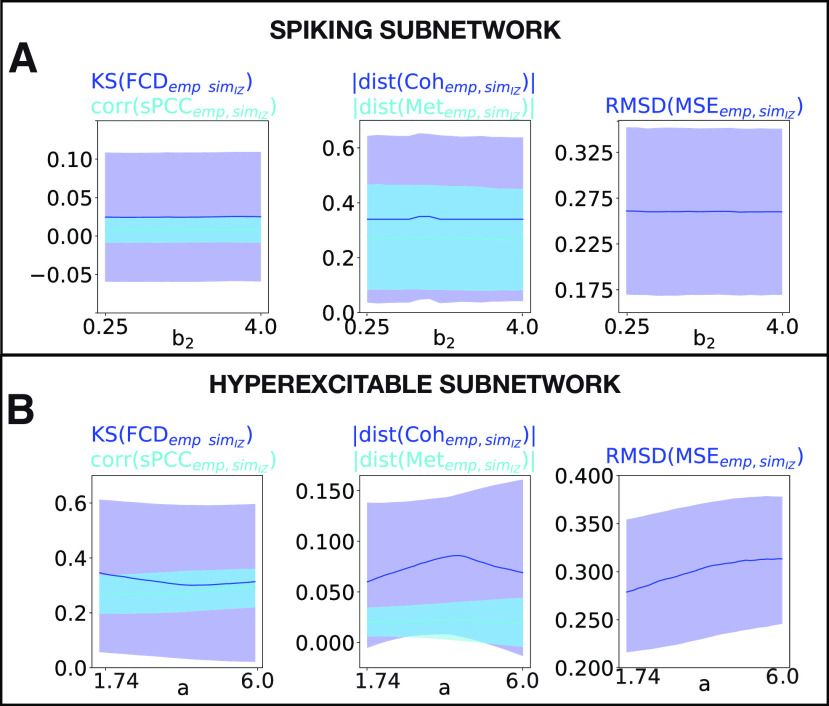
RS-FC fitting for all the epileptic patients. ***A***, ***B***, Median group of the five observables for assessing the level of fitting between empirical and simulated data are shown as a function of spike frequency *b*_2_ (***A***) and local bifurcation parameter *a* (***B***). Left column, Correlation coefficient between the empirical and simulated sPCC matrices (light curve) and the K–S distance between the empirical and simulated FCD histograms (dark curve). Middle column, Absolute value of the difference between the empirical and simulated coherence (dark curve) and metastability (light curve). Right column, RMSD between the empirical and simulated MSE profiles. Areas of faded colors represent the standard error intervals.

Finally, we assessed the level of fitting between the empirical and modeled data including the patient-specific IZ (Fig. 8*A*, median group results). The combined results for all the patients show that the measures of similarity are almost unaffected when varying the frequency of the spikes, which is in agreement with the results of the systematic analysis described above and that are shown in Fig. 6*C*.

### Perturbations of local brain dynamics in epileptogenic brain regions trigger network-wide connectivity

The alteration of the individual local dynamics in the epileptogenic brain regions is a potential mechanism that could explain the observed empirical differences in RS-FC features between the different brain regions in epileptic brains. To test this hypothesis, we conducted a computational experiment similar to that described in the previous paragraph. Specifically, based on our model in the spike-free resting-state scenario (with *p* = 0.1 for all brain regions in Eq. 19), the local bifurcation parameter *a_i_* of the epileptogenic areas was systematically modified toward the supercritical regime (i.e., self-sustained oscillations), implying a hyperexcitability of that region, to characterize changes in global and regional connectivity due to a local shift in optimal dynamics.

**Figure 9.**
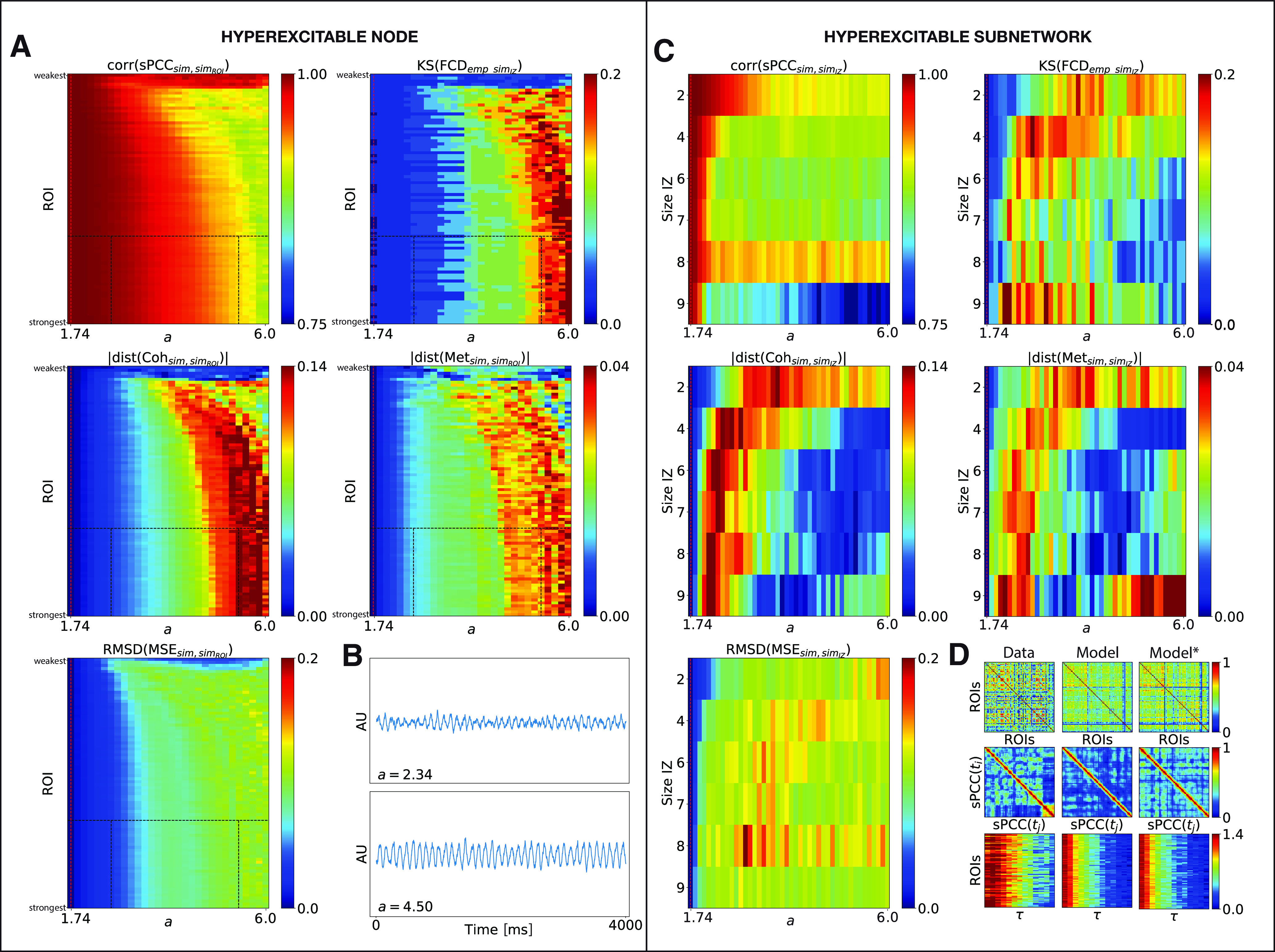
Impact of local hyperexcitability on the whole-brain RS-FC. ***A***, ***C***, The five observables for assessing changes between simulated data with and without hyperexcitable nodes in the network are shown color coded as a function of (*y*-axis) hyperexcitable region ROI sorted by structural node strength (***A***) and the size of the patient-specific hyperexcitable subnetwork IZ, and (*x*-axis) local bifurcation parameter *a* (***C***). Top row, Correlation coefficient between the two simulated sPCC matrices (left) and K–S distance between the two simulated FCD histograms (right). Middle row, Absolute value of the difference between the two simulated coherence (left) and metastability (right). Bottom row, RMSD between the two simulated MSE profiles. The red dashed lines represent the critical point. ***B***, Neural activity time series for two different values of the parameter *a* for the mid-connected brain region (***A***, black dashed lines). ***D***, Simulated sPCC, FCD, and MSE matrices for the optimal parameter *a* from the patient P1 (right). For comparison, the same matrices are plotted for the empirical data (left) and for the simulated control data (middle).

One hyperexcitable region was first introduced in the BNMs, and we examined the effect of the level of excitability *a* on the resulting simulated RS-FC values and, in particular, how this depended on the structural strength of that region. Parameter space exploration was performed by successively modifying the selected hyperexcitable region (ROI) in SC, and by varying the local bifurcation of that region. For each set of parameters (ROI, *a*), we calculated the changes in whole-brain RS-FC compared with the control case scenario (i.e., *a_i_* = *a* = 1.74, for all *i*) using the metrics defined before. The results, presented in Figure 9*A*, where the BNM was computed from the connectome of patient P1, show that a hyperexcitable node can induce fluctuations in RS-FC patterns at the whole-brain level, in particular, if this node is at least moderately anatomically connected in the network and/or for very large divergence from criticality, that is increased for higher excitability *a*, as illustrated in Figure 9*B*. We then analyzed how the sPCC connectivity of the hyperexcitable region with the rest of the brain was impacted compared with the control case. Figure 7*D* reveals a widespread significant increase in connectivity in the hyperexcitable scenario (*p* = 0.04), as the bifurcation parameter *a* diverges from criticality.

Furthermore, we investigated the impact that a hyperexcitable subnetwork could have on the RS-FC patterns, by simulating individual epileptic brain models with patient-specific IZ (including IZ1 and IZ2) replaced by a hyperexcitable zone. As shown in Figure 9*C*, changes spread faster (i.e., for smaller divergence from criticality) as the size of the network increases, although sPCC and MSE are less impacted than other metrics. Subsequently, the comparison between the sPCC connectivity of the hyperexcitable patient-specific subnetwork with other regions (Fig. 7*F*), and within the hyperexcitable subnetwork (Fig. 7*E*), to the control scenario show a significant increase in the connectivity in the hyperexcitable case (*p* = 0.04 for both panels).

Finally, we assessed the fitting between the empirical and simulated data for all the patients (Fig. 8*B*, median group results). As can be observed in Figure 8*B* (left), the correlation coefficient between the empirical and simulated sPCC matrices (Fig. 8*B*, light blue curve) is increased as the bifurcation parameter *a* diverges from criticality, and, in a similar way, the K–S distance (Fig. 8*B*, dark blue curve) decreases for higher *a*. In contrast, in Figure 8*B* (middle), the level of similarity between the empirical and simulated coherence (Fig. 8*B*, dark blue curve) slightly increases as *a* becomes higher and decreases for larger *a*; while it remains almost similar for the metastability (Fig. 8*B*, light blue curve). Regarding the complexity in Figure 8*B* (right), the difference between the empirical and simulated MSE profiles also slightly increases for larger parameter *a*. The simulated sPCC, FCD, and MSE matrices obtained at the best-fit point for patient P1 are shown in Figure 9*D* (depicted by *). For comparison, the same matrices are plotted for the empirical data (Fig. 9*D*, left), and for the control scenario (Fig. 9*D*, middle).

## Discussion

Understanding and characterizing the dynamics of epileptogenic networks within the interictal periods are critical for improving the surgical management of drug-resistant partial epilepsy. To identify the relevant variables contributing to the differences observed in brain imaging between epileptic and healthy brains, we analyzed the organization of brain network activations. These variables are physiologically unspecific, as multiple mechanisms can lead to the same behavior, but are useful in research and clinical applications. Examples include the epileptogenicity of a brain region, which maps directly between a model parameter and clinical hypothesis, and the global coupling of the brain. The former is an excitability parameter that describes the interplay between oscillations and noisy dynamics, while the latter sets the range of coactivation between brain regions and global synchrony. Using the BNM, we demonstrated that epileptic brains operated at a different global working point induced by a shift toward lower excitability and less oscillatory activity compared with healthy control subjects. We also showed that the presence of *in silico* IEDs did not modify BOLD fluctuations and connectivity patterns compared with the spike-free signals. Moreover, we established that the epileptogenic regions displayed higher excitability and more oscillatory activity, enhancing the level of similarity with the empirical data.

To this end, we extended the phenomenological neural mass model of partial seizures, Epileptor (Jirsa et al., 2014), tuned to express regionally specific physiological oscillations in addition to the epileptiform discharges. Hence, reflecting RS activity that closely resembles SEEG recordings. The extension was made using linearly coupled oscillators near a supercritical Hopf bifurcation to model the spontaneous local field potential-like signal (Ghosh et al., 2008; Deco et al., 2017a). Although the parameters of the model cannot be directly interpreted in biological terms, the degree of epileptogenicity or excitability of each brain region, characterized by the continuous control parameters *x*_0_ and *a*, was motivated by the presumed existence of the different SEEG-defined zones that have been linked to the surgical prognosis (Proix et al., 2017).

Through systematic parameter exploration and fitting to neuroimaging data, we showed that the region where the brain model fits the data best lies in the supercritical region in health, characterized by self-sustained oscillations. In epilepsy, it shifts at the edge of the bifurcation, characterized by a mixed noisy oscillatory behavior. This shift toward lower excitability implies that the neural excitation during the interictal periods free of epileptiform discharges is also affected, but opposed to the (hyperexcitable) ictal state (Scharfman, 2007). These findings are in agreement with recent studies reporting a divergence from the optimal healthy bifurcation parameter in disease, such as in schizophrenia and Alzheimer's disease (Cabral et al., 2013; Demirtaş et al., 2017). This indicates that in pathologic brains, RS emerges from a different dynamical regime compared with healthy brains. The shift may result from dynamic compensatory processes that preserve neurophysiological functioning in the presence of hyperexcitable pathologic epileptiform network activity. Other influences should be considered as well, like the effect of antiepileptic drugs, indeed, patients remained on their daily medication during the scan (Wandschneider et al., 2014) or vigilance fluctuations, as sleep is characterized by a shift toward lower excitability compared with wake RS (Jobst et al., 2017). Yet, patients are more alert than control subjects due to the higher anxiety level of the investigation. Furthermore, we demonstrated that the optimal global coupling was similar in epileptic and healthy groups. The result suggests that either the neural transmission is preserved in the disease, or region modeling of the cortex and subcortical areas does not capture the structural reorganization in epilepsy (Besson et al., 2017). This is corroborated by the nonsignificant difference found between the SC weights of the two groups. Also, it should be noted that varying the weights has less influence on the propagation activity than the topology of the SC matrix (Proix et al., 2017), demonstrating the predictive power of individual connectome-based models. Another important finding is that the best fitting of the complexity in BOLD signals using MSE was present only in a narrow range of parameters when *a* is close to the bifurcation. In other words, we demonstrated a better way of constraining BNMs, rendering the fitting process more complex and complete.

The simulation of IEDs in brain signals showed that the whole-brain RS-FC was only slightly modified when comparing to spike-free signals, and the patient-specific connectivity was almost unaffected when adding the effect of IZ. These results were independent of the spike frequency and the SC node strength of the involved areas, suggesting that the FC in epileptic brain is related to neuronal activity other than IEDs. All patients having long-standing drug-resistant epilepsies, so we can hypothesize that the epileptogenic activity may evolve from IEDs related to more permanent connectivity changes as a result of plasticity triggered by the pathologic activity, causing metabolic changes in the BOLD data. This hypothesis is corroborated by some studies showing changes over time of the size of EZ (Bartolomei et al., 2008) or alterations in SC (Bonilha et al., 2006). Our results are consistent with those of previous studies investigating connectivity patterns in the presence/absence of IEDs, and using ROI-based or ICA-based FC methods, showing that the features of the brain networks were similar in fMRI or SEEG datasets with and without IEDs (Bettus et al., 2008; van Houdt et al., 2015). We extended these results by considering the nonstationarities within the brain signals using dynamic FC and MSE analyses. We showed limited to no effect of IEDs on RS-FC changes.

The alteration of the local dynamics of the epileptogenic regions showed that whole-brain RS-FC changes to local hyperexcitability can be widespread, including regions local to and distant from the presumed focus when compared with control signals. The performance of the model was also improved, implying that the pathologic areas operate in the supercritical regime. Several works revealed similar dynamical properties by quantifying local long-range temporal (auto)correlations using detrended fluctuation analysis (Parish et al., 2004; Monto et al., 2007; Tavares et al., 2015). They suggest a persistent network abnormality due to exposure of the neuronal networks to the epileptic activity. We can then speculate that, even in the absence of visible epileptic discharges, the specific alteration of local high excitability might help to localize the epileptogenic regions in focal epilepsy by separating these areas to healthy ones, and to inform the likely outcome after surgery. Note that we had no intention to select an optimal value for each region, but rather to mimic a scenario where epileptogenic brain areas exhibited higher excitability or hyperexcitability in parallel.

To test for consistency with prior RS-fMRI findings mainly based on (linear) temporal correlation (i.e., sPCC), we compared the direction of the alterations induced in the BNMs either by the presence of IEDs or hyperexcitability within the epileptogenic networks. In line with previous work (Bettus et al., 2009, 2010; Su et al., 2015), our results showed a significant hypoconnectivity within the spiking networks, and in their connections with the rest of the brain. We also observed a significant hyperconnectivity within the hyperexcitable networks, and in their connections with the rest of the brain, corroborating empirical results (Pereira et al., 2010; Pittau et al., 2012; Wirsich et al., 2016). This supports the hypothesis that long-standing epileptic activity results in a more permanent connectivity change. Moreover, the variation of RS-FC induced by nonstationarities during the interictal RS period was recently investigated via sliding window analyses reporting both increased (Laufs et al., 2014; Pedersen et al., 2017; Ridley et al., 2017) and decreased (Nedic et al., 2015) connectivity in the epileptogenic networks. It is quite probable that these discrepancies within the same modality could be explained by the different methodologies used or also by the vigilance fluctuations that affect RS-FC estimates (Tagliazucchi and Laufs, 2014).

Note that the results obtained in this study are constrained by the modeling framework that, apart from the level of epileptogenicity, keeps all the other parameters constant across brain regions. In this way, while capturing some basic biological principles, and especially those that cause interictal discharges or higher excitability of the epileptogenic regions, we are likely to miss some system features. Regional heterogeneity of the parameters of the computational models has already proven to improve the fit of the FC (Deco et al., 2019; Wang et al., 2019). However, without a clear physiological prior to the regional distribution of excitability or some other dynamical or biological parameter, there is a risk to overfit the data. Hence the current approach, which in the future could be improved using parameters obtained by Bayesian inference or other fitting techniques (Deco et al., 2017a; Jirsa et al., 2017; Hashemi et al., 2020), would go beyond epileptogenicity.

Placed in a personalized BNM framework, the novel hybrid model combines generic network dynamic features of neuronal populations at rest and during seizures with patient-specific (compared with BNM informed by generic assumptions or average brains) predictive power of measured network connectivity, that is accessible in human brain imaging. Therefore, despite the loss of direct causality of biophysical mechanisms, the approach advocated here is more explanatory due to its general nature than if it were generated using detailed mechanistic models.
